# The Role of Invariant Natural Killer T Cells in Dendritic Cell Licensing, Cross-Priming, and Memory CD8^+^ T Cell Generation

**DOI:** 10.3389/fimmu.2015.00379

**Published:** 2015-07-28

**Authors:** Catherine Gottschalk, Elisabeth Mettke, Christian Kurts

**Affiliations:** ^1^Institute of Experimental Immunology, Rheinische Friedrich-Wilhelms-University of Bonn, Bonn, Germany

**Keywords:** natural killer T cells, dendritic cells, licensing, memory, CD8 T cells, cross-presentation

## Abstract

New vaccination strategies focus on achieving CD8^+^ T cell (CTL) immunity rather than on induction of protective antibody responses. While the requirement of CD4^+^ T (Th) cell help in dendritic cell (DC) activation and licensing, and in CTL memory induction has been described in several disease models, CTL responses may occur in a Th cell help-independent manner. Invariant natural killer T cells (iNKT cells) can substitute for Th cell help and license DC as well. iNKT cells produce a broad spectrum of Th1 and Th2 cytokines, thereby inducing a similar set of costimulatory molecules and cytokines in DC. This form of licensing differs from Th cell help by inducing other chemokines, while Th cell-licensed DCs produce CCR5 ligands, iNKT cell-licensed DCs produce CCL17, which attracts CCR4^+^ CD8^+^ T cells for subsequent activation. It has recently been shown that iNKT cells do not only enhance immune responses against bacterial pathogens or parasites but also play a role in viral infections. The inclusion of iNKT cell ligands in influenza virus vaccines enhanced memory CTL generation and protective immunity in a mouse model. This review will focus on the role of iNKT cells in the cross-talk with cross-priming DC and memory CD8^+^ T cell formation.

## Classification of Natural Killer T Cells

Natural killer T cells (NKT cells) are a subset of lymphocytes with innate and adaptive immune functions, for example, in tumor and anti-infectious defense (1). Their TCR can be either semi-invariant and encoded by a germline Valpha gene [type I invariant natural killer T cells (iNKT cells)] or may react against the self-antigen sulphatide using an oligoclonal TCR (type II NKT cells) (2–4). This review focusses on iNKT cells in dendritic cell (DC) licensing and T cell activation leading to a sustained memory response.

Invariant natural killer T cells respond to the marine sponge (*Agelas mauritianus*)-derived glycolipid alpha-galactosylceramide (αGalCer) presented by the non-polymorphic CD1d molecule and respond by rapidly producing various cytokines (5, 6). Mostly studied in mice, they represent about 0.5% of T cells in the blood, 2% in secondary lymphatic organs, and over 30% of T cells in the liver. During inflammation and infection, iNKT cell numbers can strongly increase in numerous organs, e.g., the pancreas in type I diabetes or the lung in asthma (7, 8). In human blood, only 0.1–0.2% of T cells are iNKT cells, with 5× lower numbers than in mice (9). Recently, iNKT cells came into focus as promising targets for the development of vaccine adjuvants and immunotherapies, mostly in the field of cancer treatment and in autoimmune and inflammatory diseases (Table 1). Preclinical studies using αGalCer demonstrated moderate therapeutic activity by activating DCs and providing Th-like functions, generating CD8^+^ cytotoxic T cell (CTL) and antibody responses. Currently, more potent αGalCer analogs for iNKT cell activation are under investigation (10–13). Applying NKT cell immunization schemes in clinical settings is a promising therapeutic opportunity, but requires detailed knowledge on how iNKT cells activate DCs.

**Table 1:** **Summary of iNKT cell activation studies in treatment of different diseases**.

Therapeutic target	Species studied	Outcome	Reference, remarks
Viral and bacterial infections	Human, mouse	Effective vaccination in mice; oral and nasal route possible; no clear effect on chronic viral infections in clinical trials shown	(12–21)
Parasites and fungi	Mouse	Enhanced vaccine effects in mice	(10, 22–24) αGalCer analogs were used in Ref. (10) (7DW8–5) and Ref. (20) (α-C-GalCer) for NKT cell activation
Tumors	Human, mouse	Enhanced tumor protection and rejection in mice; clinical trials show only moderate effects in humans	(11, 14, 24–39) Antigen-pulsed DC were transferred in Ref. (32), no αGalCer or analog was added. αGalCer and α-C-GalCer were tested for tumor therapy in Ref. (34)
Autoimmune diseases	Mouse	αGalCer dose-dependent amelioration or aggravation of autoimmune diseases; NKT cell hypo-responsiveness involved in some cases	(8, 40–52) Ref. (43) used OCH, a sphingosine-truncated analog of αGalCer for NKT cell activation

*iNKT cells were activated by αGalCer treatment if not indicated otherwise*.

## iNKT Cell Activation, Subsets, and Cytokine Production

Most knowledge on NKT cell activation came from the use of αGalCer, a strong and prototypical CD1-restricted agonist. In the last years, additional microbial-derived glycolipid ligands were identified, including α-glucuronosylceramides (from *Sphingomonas*), cholesteryl α-glucoside (from *Helicobacter*), or diacylglycerol-containing glycolipids (from *Borrelia*) (53, 54). These lead to sustained iNKT cell activation with inflammatory cytokine production that is independent of TLR stimulation, IL-12, or the recognition of endogenous antigens, hence relying only on engaging the invariant TCR. α-glucuronosylceramide induces IFNγ and IL-4 release similar to αGalCer (55–57). Both glycolipid antigens are structurally similar and can be recognized by the majority of mouse and human iNKT cells (58). Synthetic iNKT cell antigens have been and continue to be studied extensively for potential therapeutic application (59). However, iNKT cell activation may also promote allergic airway inflammation, and their overstimulation can induce iNKT cell anergy (1, 60).

Most microorganisms lack cognate iNKT cell antigens, hence activation of these cells relies on cytokines, such as IL-12 or IL-18, in conjunction with endogenous antigens. Even in the absence of TCR stimulation, some bacterial and viral infections induce a robust IL-12 response by DCs thereby activating iNKT cells *in vivo* (61, 62). Indirect iNKT cell activation results in the release of IFNγ but usually not IL-4 and is not restricted to TLR (62–65).

Analogous to Th cells subsets, different NKT cell subsets termed NKT1, NKT2, NKT17, NKT_FH_, and NKT10 subsets were described with corresponding functionalities (66, 67). NKT17 cells produce the cytokines, IL-17 and IL-22, and are abundant in the lymph nodes, lungs, and skin of mice with airway neutrophilia induced by αGalCer (68). Recently, it was shown that iNKT17 cells are enriched in NOD mice, a mouse model for type I diabetes, which hint toward a possible role of those cells in disease development (69). iNKT17 cells rely on IL-7 for homeostasis and survival (70) and seem to require activation in the presence of TGF-β and IL-1β (71). The recently described NKT10 subset can dampen inflammatory responses by IL-10 production and is enriched in adipose tissue, providing protection in obesity-induced inflammation (72).

## Dendritic Cell Maturation and CD8^+^ T Cell Cross-Priming

Dendritic cells classically gather antigens in tissues and transport them into lymphatic organs, where they orchestrate the activation and differentiation of naïve CD8^+^ T cells into CTL. Recent work showed that some DCs remain in tissues in order to regulate immigrating effector T cell responses, which is important in the defense against infections and may also promote the progression of many immune-mediated diseases. The cross-talk of myeloid cells with other immune cells, such as T cells and innate lymphocytes, is especially important in this context. Cellular encounters are orchestrated by chemokines, cytokines, and cell surface molecules. Some DCs, especially the XCR1^+^ DC subset, are specialized in cross-presentation, which allows the presentation of extracellular antigens to activate CTL, a process important for immunity against tumors, viruses, and intracellular bacteria and for vaccination (73–76). Immunogenic cross-presentation, also referred to as cross-priming, requires the presence of pathogen-derived molecules (PAMPs) and/or of specific Th cells or NKT cells that mature the cross-presenting DC (77). This process is called “licensing,” a term introduced by Lanzavecchia (78), and it aims at preventing unwanted immune answers against innocuous or self antigens. Licensing was first described by Matzinger, Heath, and Melief (79–81), and classically is mediated by CD40 ligand provided by specific CD4^+^ helper T cells (Th). In addition to licensing, immunogenic T cell priming requires the DCs to mature, a process that results from sensing various PAMPs, including ligands for TLR, lectins, intracellular nucleotide-binding oligomerization domain receptors, or retinoic acid-induced genes (82–85). Major consequences of DC maturation are the upregulation of costimulatory molecules like CD80 and CD86, CD40, of MHC II and the production of pro-inflammatory cytokines, especially IL-12p70 and TNF. These consequences partially can result also from CD40–CD40L interactions, but it is not clearly defined how much DC licensing and maturation functionally overlap. CD40–CD40L interactions are not only crucial for upregulation of costimulatory molecules but also for DC survival (86). Additionally, mature DCs produce chemokines to attract other immune cells and to orchestrate the ongoing immune response. In contrast to maturation-induced upregulation of MHC II, CD1 trafficking is differentially regulated during DC maturation, and CD1 molecules are already expressed on immature DCs. While human DCs express all classes of CD1 molecules, murine DCs express only CD1d (87), which is crucial for DC–iNKT cell interactions. Trafficking studies showed that antigen presentation by CD1d to iNKT cells might already occur before DC maturation and MHC II presentation (88). This notion hinted to a possible role of iNKT cells as immunological helper cells.

## iNKT Cells as Immunological Helper Cells

αGalCer was found to mediate CD40-dependent activation of CTL by NKT cell-helped DC (89), directing attention to the adjuvant activity for this agent. Furthermore, αGalCer also induced resistance to tumors and intracellular pathogens (25). Compared to CD40 ligation, LPS, and CpG, αGalCer induced equally high levels of CD40, CD80, CD86, MHC II, and DEC205 in CD11c^+^ CD8a^+^ and CD11c^+^ CD8a^−^ DCs, but was unable to induce DC maturation from bone marrow progenitors. Rather than acting directly on DCs, αGalCer mediated DC maturation through iNKT cells in a MyD88-independent manner. Combining αGalCer with CD40 stimulation caused DC to produce high amounts of IL12p70, while LPS and CD40 stimulation showed no such effect. IL12p70 production might explain the results of another study (90), where the simultaneous administration of OVA and αGalCer enhanced Th and CTL responses in an iNKT cell-dependent manner. A close temporal association between αGalCer and OVA-derived peptides and additional experiments with antigen-loaded DCs led to the conclusion that αGalCer and peptides must reach the same DC. Formal *in vivo* evidence for such a tripartite cellular interaction was provided with the use of bm1/CD1d bone marrow chimeras (91). In addition, there was synergy when Th and iNKT help were combined. The means by which iNKT cells license DCs are not fully understood but in addition to providing CD40L to DCs, iNKT cells may act by promoting cross-talk of XCR1^+^ DCs and plasmacytoid DC (92) or by abundant cytokine production upon activation. Whether iNKT cells play a role as helper cells when activated by less potent ligands remains to be elucidated.

## iNKT Cells Help in CTL and CD8^+^ Memory T Cell Formation

**Figure 1:**
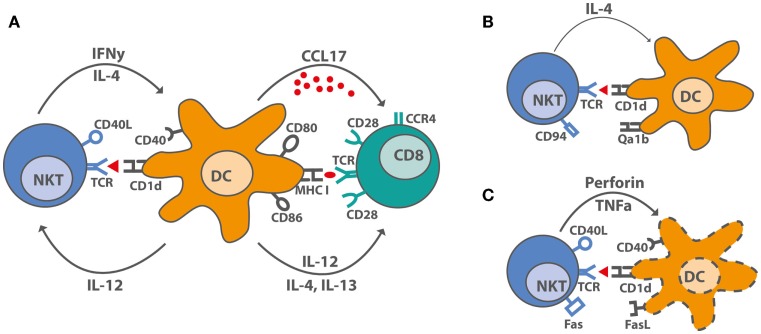
**iNKT cell–DC interactions after stimulation with αGalCer**. **(A)** Under optimal stimulatory conditions, iNKT cells produce IL-4, large amounts of IFNγ and upregulate CD40L, thereby inducing maturation in DC. DC maturation leads to increased costimulatory capacity through upregulation of CD80 and CD86, of MHC molecules, and by producing the pro-inflammatory cytokine, IL-12, and the chemokine, CCL17. CCL17 attracts CCR4^+^ cells, including CD8^+^ T cells, which can be activated by the licensed DC. **(B)** Overstimulated iNKT cells upregulate inhibitory receptors like CD94 and are incapable of producing IFNγ. DC interacing with hyporesponsive iNKT cells cannot be activated and do not induce CD8^+^ T cell activation. **(C)** Some activated iNKT cells induce DC lysis rather than maturation by yet unknown mechanisms. Proposed mechanisms suggest a role for TNFα, perforin, Fas–FasL interactions, and even CD40–CD40L.

The knowledge on mechanisms iNKT cells use to substitute CD4^+^ T cell help for antibody production, CTL generation, or memory formation is central for developing new vaccination strategies. An unresolved question is why some groups observed NKT cell-dependent reduction of CTL-mediated autoimmune diseases, whereas NKT cell-licensed DC induced strong CTL responses against tumors and viruses in other studies. The most obvious difference is the use of a single low dose of αGalCer for induction of protective CTL responses and the use of multiple doses or high single doses of αGalCer to inhibit unwanted T cell responses (93). In some clinical trials, αGalCer was used to treat cancer, and human CD4^+^ iNKT cells expanded predominantly during early stages (26). CD4^+^ iNKT cells can induce IL12p70 production by DC and thereby Th1 polarization (93). Double negative (DN) iNKT cells expanded later after αGalCer treatment and can induce apoptosis in αGalCer-loaded DC, thereby limiting the immune response (26). Functional differences between iNKT cell subsets in regards to cytokine production are evident both in mice and humans (94), but the effects on DC maturation, apoptosis, and CTL generation remain to be elucidated (Figure 1). A high frequency of a DN iNKT cell subset and their potential to lyse DCs may impair treatment of cancer patients and vaccination strategies. The role of iNKT cells during viral infections and the use of αGalCer as vaccine adjuvant in the context of influenza infections have been reviewed recently in Ref. (14, 95). αGalCer increased the levels of influenza-specific systemic IgG and mucosal IgA antibodies, even in the absence of Th cells and antigen-specific CTL responses (14, 95). In contrast, after combined iNKT cell activation and influenza virus vaccination, an impaired CTL response but enhanced memory CTL generation was seen (96). In line with this, enhanced CTL memory differentiation during viral infection was also shown previously (97). Another study showed that iNKT cell enrichment in the CNS during Theiler’s murine encephalomyelitis virus (TEMV) infection inhibited the antiviral CTL response and delayed the accumulation of TEMV-specific CTL. Also, the magnitude of the TEMV-specific CTL response was impaired (98). CTL memory formation was not assessed in that study. Co-administration of αGalCer with suboptimal doses of irradiated sporozoites or recombinant viruses expressing a malaria antigen enhanced protective anti-malaria immunity in mice, and co-administration of αGalCer with various immunogens enhanced antigen-specific CTL responses and Th1 responses (99). In conclusion, vaccination with αGalCer as adjuvant induced iNKT cell help for DCs, which promoted CTL memory formation but impaired primary antigen-specific CTL responses.

Before we can fully understand the mechanisms of iNKT cell help in CTL formation and memory generation, it is crucial to know how “help” influences CTL responses in general. Many reports about Th help are available and most of them show a diverse picture of the requirement for CD4^+^ T cell help in primary and/or secondary infections. Th help seems to be crucial for the clearance of some primary virus infections like HSV or influenza that do not affect DCs directly (100, 101), while in some viral infections, Th help can be overcome (102). Additionally, CTL responses against minor H antigens, soluble proteins, tumors, and peptide-pulsed DC require Th help for the induction of optimal primary responses (79, 103, 104). Some groups disagreed whether Th help was needed during secondary responses for proper re-expansion of CTL (105, 106) or whether Th help was merely a prerequisite during primary infections for CTL memory formation (107). Additionally, Th help was dispensable for the expansion, but not for the cytotoxic capacity of CTL in tuberculosis (108).

These differential observations may be explained not only by the variance of pathogens and model antigens used but also by different experimental setups. Moreover, Th dependency was studied by using CD4^−/−^ mice, MHC II^−/−^ mice, or CD4-depleting antibodies, which are not biologically equivalent (109). For example, CD4-depleting antibodies also deplete regulatory T cells, CD4^+^ NKT cells, and CD4^+^ DCs. However, most older studies agreed that the requirement for Th help is not a CTL-intrinsic property but dependent on the infectious agent and DC maturation. Given the huge discrepancies in studies on Th help requirements, observations in a single model do not permit general conclusions on how CD4^+^ help may be substituted by iNKT cells. A deeper insight into CTL generation and memory formation is required to allow predictions for the role of iNKT cell help in CTL responses.

As reviewed previously in Ref.(110), CTL in primary responses can be divided into short-lived effector cells (SLEC) that mostly die off during the contraction phase and memory precursor cells (MPEC) that received less stimulation but more survival signals (111, 112). Even a single naive CTL can differentiate into a diverse population of effector and memory cells (113, 114) by multiple mechanisms, which have been reviewed in detail by Kaech and Cui (115). Prolonged antigen exposure and pro-inflammatory cytokines like IL-12 and IL-2 promote terminal differentiation of CTL and induce superior cytotoxic capacities (116–118). NKT cells may affect CTL differentiation other than Th cells, but this hypothesis requires further experimental exploration.

## iNKT Cells and Chemokines in CTL and CD8^+^ T Cell Memory Formation

Chemokines play a major role in orchestrating primary and memory CTL responses. During infections, CTL upregulated CXCR3, which allowed them to enter peripheral tissues (119). Th help was required for enhanced recruitment of CTL to the site of infection in some situations (120) by promoting CXCL9 and CXCL10 production, with CXCL9 being especially important for rapid memory responses in the lymph node (121). Infections of the lung and intestine showed no requirement of Th help for migration as lung infections, e.g., by influenza induce on-site proliferation of CTL rather than recruitment (122, 123) but Th cells promoted development of lung-resident memory cells (124).

CXCR3 also drove CTL toward an effector fate rather than memory fate (125). In line with this, CXCR3 × CCR5 double-deficient mice showed a decreased contraction phase and harbored more memory CTL, which were unable to migrate into tissues and to clear infections (126). In humans, CCR5 expression was associated with effector memory T cells, whereas CCR7 was predominantly expressed on naïve and central memory T cells and CCR6 expression was found on early effector memory T cells (127–129).

iNKT cell-helped DCs produced high amounts of CCL17, thereby attracting CCR4^+^ lymphocytes (91). This contrasts the situation in classical Th cell-dependent cross-priming, where DCs produced CCR5 ligands to attract CTL for cross-priming. These chemokines synergically guided CTL toward those DCs that have presented relevant antigen to helper T cell subsets, and thereby facilitated the ensuing CTL response. Thus, CCR4- and CCR5-binding chemokines have been described as a new signal in T cell activation, distinct from signal 1, antigen, and signal 2, costimulation (130).

CCR4 is traditionally considered to be associated with skin homing Th2 and memory CD4^+^ T cells (131–134), but also with the recruitment of Treg to the inflamed liver (135). Several studies in humans showed increased CCR4 expression also on CTL in cutaneous diseases (136–138). A CCR4^+^ CD8^+^ central memory subset has been described that was generated in the presence of IL-4 and produced IL4 and IL-13 upon restimulation (139). These cells were not cytotoxic and produced little IFNγ, features associated with a so-called Tc2 subset (139, 140). Kondo and Takiguchi showed that human CCR4^+^ CD8^+^ T cells expressed less effector molecules like perforin or granzymes compared to CCR6^+^ early effector memory T cells, but produced more TNFα and IL-4 than CCR7^+^ naïve or central memory CD8^+^ T cells. They concluded that CCR4^+^CD8^+^ T cells are a “little more differentiated than CCR7^+^ central memory ones and less differentiated than CCR6^+^ early effector memory ones” and that they can migrate into secondary lymphoid organs where they mature after interacting with DCs expressing CCR4 ligand (141). Since iNKT cells produce IL-4 upon activation and induce CCL17 production by helped DC, they might play a role in the development or restimulation of CCR4^+^ CD8^+^ T cells. The physiological role of this subset in viral infections and tumors remains to be elucidated.

## Concluding Remarks

CD4-helped DCs and NKT cell-helped DCs provide various costimulatory signals and cytokines deciding the fate of CD8^+^ T cells toward effector or memory. However, the set of chemokines produced by NKT cell-helped DCs attract different subsets of naïve or memory CD8^+^ T cells compared to chemokines produced by Th-helped DCs. Dissecting the role of those CD8^+^ T cells subsets in effector and memory responses directed against tumors and viral infections may facilitate developing effective NKT cell-based vaccines.

## Conflict of Interest Statement

The authors declare that the research was conducted in the absence of any commercial or financial relationships that could be construed as a potential conflict of interest.
